# Optimizing Outcomes in Cardiac Rehabilitation: The Importance of Exercise Intensity

**DOI:** 10.3389/fcvm.2021.734278

**Published:** 2021-09-03

**Authors:** Jenna L. Taylor, Amanda R. Bonikowske, Thomas P. Olson

**Affiliations:** Division of Preventative Cardiology, Department of Cardiovascular Medicine, Mayo Clinic, Rochester, MN, United States

**Keywords:** interval training, coronary artery disease, cardiovascular disease, heart failure, cardiorespiratory fitness, peak oxygen consumption, exercise prescription, progression

## Abstract

Exercise based cardiac rehabilitation (CR) is recognized internationally as a class 1 clinical practice recommendation for patients with select cardiovascular diseases and heart failure with reduced ejection fraction. Over the past decade, several meta-analyses have generated debate regarding the effectiveness of exercise-based CR for reducing all-cause and cardiovascular mortality. A common theme highlighted in these meta-analyses is the heterogeneity and/or lack of detail regarding exercise prescription methodology within CR programs. Currently there is no international consensus on exercise prescription for CR, and exercise intensity recommendations vary considerably between countries from light-moderate intensity to moderate intensity to moderate-vigorous intensity. As cardiorespiratory fitness [peak oxygen uptake (VO_2_peak)] is a strong predictor of mortality in patients with coronary heart disease and heart failure, exercise prescription that optimizes improvement in cardiorespiratory fitness and exercise capacity is a critical consideration for the efficacy of CR programming. This review will examine the evidence for prescribing higher-intensity aerobic exercise in CR, including the role of high-intensity interval training. This discussion will highlight the beneficial physiological adaptations to pulmonary, cardiac, vascular, and skeletal muscle systems associated with moderate-vigorous exercise training in patients with coronary heart disease and heart failure. Moreover, this review will propose how varying interval exercise protocols (such as short-duration or long-duration interval training) and exercise progression models may influence central and peripheral physiological adaptations. Importantly, a key focus of this review is to provide clinically-relevant recommendations and strategies to optimize prescription of exercise intensity while maximizing safety in patients attending CR programs.

## Introduction

Exercise-based cardiac rehabilitation (CR) is a class 1A recommendation for patients with select cardiovascular diseases (CVD) and heart failure with reduced ejection fraction (HFrEF), as it leads to significant improvements in exercise capacity, CVD risk profile, and reductions in hospital readmissions, cardiovascular (CV) events, and mortality (1–6). Compared with standard medical care, systematic reviews from 2004 (5) to 2011 (4) in coronary heart disease, showed that exercise-based CR reduced hospitalizations, CV mortality, and all-cause mortality by 31, 26, and 20 %, respectively (4, 5). However, over the past decade, results from RAMIT (Rehabilitation after Myocardial Infarction Trial) (7) and subsequent systematic reviews (8, 9) questioned the effectiveness of exercise-based CR for reducing recurrent CV events (7, 9), CV mortality (9), and all-cause mortality (7–9).

This generated substantial debate within the scientific community (10–12), with speculation that low exercise training intensity and dose may be responsible (13). Moreover, meta-analyses have shown significant heterogeneity and lack of detail regarding exercise prescription methodology within CR programs (2, 3). Currently there is no international consensus on exercise prescription or program duration for CR, and exercise intensity recommendations vary considerably between countries from light-moderate intensity (Australia, Japan), moderate intensity (United Kingdom, France), and moderate-vigorous intensity (Canada, United States, South America, and other European countries) (14). Furthermore, studies from the United Kingdom have highlighted that in CR practice the actual exercise training intensities performed by patients, may not progress to the upper range of exercise intensity recommendations (13). Despite numerous publications outlining international CR practices and program characteristics from national registries or surveys, relatively few include data on exercise intensity prescription or implementation (15–18). This data is important to determine whether exercise training during CR is being prescribed and implemented effectively across international programs. There is a need to define internationally accepted standards in CR delivery and scientific evaluation (2, 3).

This review will examine the evidence for prescribing moderate-vigorous intensity aerobic exercise and high intensity interval training (HIIT) in CR programs, including beneficial physiological adaptations to the pulmonary, cardiac, vascular, and skeletal muscle systems in patients with CVD and heart failure (HF). Moreover, this review will discuss how increasing the duration of intervals and training volume may improve physiological adaptations; and will discuss practical applications and progression models to optimize exercise prescription in CR programs.

### Exercise Prescription in Cardiac Rehabilitation

Methods for prescribing exercise intensity in CR vary internationally but can also be program-specific depending on the resources available. Objective methods for determining exercise intensity can include indices of peak exercise capacity, ventilatory thresholds, anaerobic threshold, or the myocardial ischemia threshold. These require availability of maximal exercise testing, preferably with cardiopulmonary gas analysis for intensities based on peak oxygen uptake (VO_2_peak), and/or ventilatory thresholds. In programs where maximal exercise testing is not available, subjective measures of exercise intensity including rating of perceived exertion (RPE) (19) or the talk test (TT) (20), are predominately used to guide exercise intensity.

#### Indices of Peak Exercise Capacity

The majority of guidelines on exercise training in CR recommend aerobic exercise prescription based on relative indices of peak exercise capacity. These include percentage of peak workload (Wpeak), percentage of peak heart rate (%HRpeak), percentage of VO_2_peak (%VO_2_peak), percentage of HR reserve (%HRR), or percentage of VO_2_ reserve (%VO_2_R) (1, 21). Reserve calculations are generally preferred for precise exercise intensity prescription given they also take into account the patient's resting values (22) and may be more appropriate for patients with chronotropic incompetence (23). In addition to issues with practicality of maximal exercise testing (due to cost, lack of expertise, technological resources, and/or medical supervision) (24, 25), limitations with using relative indices of peak exercise capacity can include patient failure to reach a near-maximal effort, subsequent dose adjustment and timing of rate-control medications, and the fact that VO_2_peak or Wpeak are highly influenced by the ramp rate during the test (1, 26). Furthermore, a disadvantage with a workload-based approach is that progression is based on arbitrary increments, rather than a physiological change with improvements in exercise capacity (as HR does) (1).

#### Ventilatory Thresholds

An alternative approach to using indices of peak exercise capacity, is to relate exercise intensity to ventilatory thresholds. This approach requires cardiopulmonary gas analysis and is more commonly used for exercise prescription in European CR programs. Nomenclature of these thresholds remains controversial and methodologies to assess them are not universally accepted. The first ventilatory threshold (VT1) (*also termed anaerobic threshold*) is most widely known and represents the transition from a predominately aerobic metabolism to a point where blood lactate begins to accumulate and a greater reliance on anaerobic metabolism is needed for continued energy production (1, 27). At this point, ventilation (VE) accelerates to counterbalance and eliminate the excess carbon dioxide (CO_2_) in the blood produced during the conversion of lactic acid to lactate (27). The second ventilatory threshold (VT2) (*also termed respiratory compensation point, critical powe*r, *or lactate threshold*) represents the exercise intensity at which blood lactate accumulates rapidly, excess CO_2_ can no longer be eliminated, and there is a disproportionate increase in VE relative to CO_2_ production (VCO2) (1, 28). The VT1 is commonly assessed using the V-slope method (i.e., the departure of VO_2_ from a line of identity drawn through a plot of VCO_2_ vs. VO_2_) or the nadir (lowest point) of the VE/VO_2_ to work rate relationship (1, 27). The VT2 is assessed as the nadir of VE/VCO_2_ to work rate relationship (1, 28). Exercise training zones can then be extrapolated from these thresholds using a corresponding HR or workload, with light intensity below VT1, moderate intensity between VT1 and VT2, and high intensity above VT2 (1). There are several disadvantages with using threshold-based exercise prescription. There can be substantial within-subject variability from two consecutive tests (29), a high variation between observers and sites (30), and the reproducibility of VT2 is not well established in patients with CVD (1). Furthermore, VT thresholds cannot be directly translated to constant-load exercise due to slowed VO_2_ kinetics and delay in VO_2_ response to the imposed work (1, 31), which is exaggerated in patients with CVD and HF (32–34).

#### Subjective Measures

Regardless of whether objective measures of effort (such as HR or VO_2_) are available, subjective measures of effort (e.g., RPE or TT) should be used as an adjunct in CR settings, particularly for patients who have difficulty obtaining a reliable or meaningful exercise-related HR (e.g., patients with atrial fibrillation, pacemakers, chronotropic incompetence, heart transplant, or patients receiving beta blockade therapy) (23, 28). Subjective measures can also be useful for comparing the perceived effort across exercise modalities (28). The Borg 6–20 RPE scale is a widely used instrument to measure exercise intensity, by asking patients to self-report their perceived effort of exercise on a scale of 6 (no exertion at all) to 20 (very, very hard) (19). It is a practical, validated, and effective method for prescribing and monitoring exercise intensity in patients with CVD (35, 36) and HF (37, 38), and is not influenced by beta-blocker medication (38). Limitations of using the RPE scale may include the influence of psychological factors or environmental conditions (28), difficulties in patients with impaired vision (23), or use during outdoor exercise (24). Lack of familiarity with exercise training, fitness level, age, gender, education level, and use of diuretics have also been reported to influence RPE (39–41). It is imperative that patients are educated on correct use of the RPE scale, anchored to sensations of *extremely hard/maximal* and *no exertion at all*, and representing an integrated rating of muscular and cardiovascular sensations (19, 26). The TT is another practical tool for prescribing exercise intensity, that has shown to be valid and reliable in patients with CVD (20). Physiologically, it is based on the swift increase in breathing above VT2 (or lactate threshold) that causes difficultly in comfortable talking during exercise (1), and can therefore help to identify the boundary between moderate and vigorous intensity exercise (24). The TT is not a practical tool for customizing interval training protocols with short durations (<1 min) or at very high intensity (>95 %HRpeak) (20), however further research into its use for longer duration HIIT protocols of 85–95 %HRpeak [e.g., 4 × 4 min protocol (26, 42)] would be of interest. For home-based HIIT, Wisløff et al. instructed patients with HF to complete a 4 min interval at an intensity where “they are breathing heavily and talking becomes uncomfortable” (43), which corresponded to an RPE of 17 ± 1 and 93 ± 3 % of HRpeak (44).

#### Summary

Recent guidelines have suggested that while subjective measures can be a practical method to prescribe exercise intensity, they should be used as an adjunct rather than alternative to objective methods (1). Furthermore, concerns have been raised that exercise training intensity based on results of indirect, submaximal exercise testing (e.g., 6 min walk test, incremental shuttle walk test), which do not rigorously evaluate the cardiorespiratory system, may result in under-prescription of exercise intensity and reduced effectiveness of CR programs (12). Where available, VO_2_peak and HRpeak should be determined from a maximal cardiopulmonary exercise test, during which the patient has taken prescribed HR-modulating medications. If a maximal exercise test is not feasible, a new predictive equation combining age and HR measured during a 200 m fast walk test (45), has shown good correlation with HRpeak measured during a maximal exercise test. In this case, accounting for age and HR response to a submaximal test may be more predictive of an accurate HRpeak than relying on age-predicted equations for patients with (46) and without beta-blockade (47, 48). However, the equation for patients with beta-blockade (46) accounts for resting HR and test mode in addition to age (46).

### Is There a Benefit for Prescribing Higher Intensity Exercise?

#### Definition of Exercise Intensity Ranges and Protocols

Classifications of exercise intensity by the American College of Sports Medicine (ACSM) (49) and European Association of Preventative Cardiology (EAPC) (1) are outlined in Table 1. As exercise performed at a vigorous to high intensity cannot be sustained for long periods, HIIT can be a more feasible method by alternating bouts of high intensity exercise with recovery bouts of lower intensity exercise or no exercise. There has been a large amount of scientific interest regarding HIIT in patients with CVD and HF, mostly comparing its effectiveness to moderate intensity continuous training (MICT). Sprint interval training (SIT) involves intense “all-out” or supramaximal efforts (i.e., workloads greater than VO_2_peak or peak power output) with typically shorter bouts (<45 s) (50). Although HIIT involves near-maximal intensities, efforts are still submaximal (i.e., workloads below VO_2_peak or peak power output), and therefore HIIT has been considered more appropriate for use in clinical populations than SIT (51). The terminology of HIIT and MICT are preferred given they provide a description of intensity (51). However, aerobic interval training (AIT) and aerobic continuous training (ACT), respectively are alternative terminology frequently used within with the literature. Common intensity prescription of HIIT and MICT used in patients with coronary artery disease (CAD) and HF are outlined in Table 2, devised from studies included in reviews by Pattyn et al. (52) and Taylor et al. (53). While these ranges outline the HIIT and MICT prescriptions for the majority of studies in cardiac patients, two studies have prescribed notably higher intensities for MICT in CAD including 60–80 %VO_2_peak (54, 55) and 65–85 %HRpeak (56). There are also three studies that prescribed notably lower intensity for HIIT, with one in CAD (50 % peak workload from a steep ramp test) (57), and two in HF with 50–80 % maximal power (58) and 50–75 % of VO_2_peak (59).

**Table 1:** Classification of aerobic exercise intensity.

**ACSM Guidelines (** **49** **)**			**EAPC and ESC Guidelines (** **1** **)**			
**Intensity**	**VO_**2**_ and HR**	**RPE**	**Intensity**	**VO_**2**_ and HR**	**RPE**	**Training zone**
Light	37–45 %VO_2_max 57–63 %HRmax 30–39 %HRR	9–11	Low	<40 %VO_2_max	10–11	Aerobic
				<55 %HRmax		
				<40 %HRR		
Moderate	46–63 %VO_2_max 64–76 %HRmax 40–59 %HRR	12–13	Moderate	40–69 %VO_2_max	12–13	Aerobic
				55–74 %HRmax		
				40–69 %HRR		
Vigorous	64–90 %VO_2_max	14–17	High	70–85 %VO_2_max	14–16	Aerobic + lactate
	77–95 %HRmax			75–90 %HRmax		
	60–89 %HRR			70–85 %HRR		
Near-maximal to maximal	>90 %VO_2_max	>17	Very high	>85 %VO_2_max	17–19	Aerobic + lactate + anerobic
	>95 %HRmax			>90 %HRmax		
	>89 %HRR			>85 %HRR		

*Adapted from guidelines from the American College of Sports Medicine (ACSM) (49) and European Association of Preventative Cardiology (EAPC) (1). HR, heart rate; HRR, heart rate reserve; RPE, rating of perceived exertion on 6–20 Borg scale (19); VO_2_, oxygen uptake*.

**Table 2:** Common intensity prescriptions for HIIT and MICT.

	**Patients with CAD**		**Patients with HF**	
**Training protocol**	**HR or VO_**2**_**	**Other measures**	**HR or VO_**2**_**	**Other measures**
MICT	60–75 %HRpeak	RPE 11–14	60–75 %HRpeak	50–75 %PPO
	60–85 %HRR	50–65 %PPO	45–60 %HRR	90–100 %VT1
	50–60 %VO_2_peak	100–110 %VT1	60–70 %VO_2_peak	
HIIT	80–100 %HRpeak	RPE 15–18	80–95 %HRpeak	90–100 %PPO
	80–95 %HRR	90–110 %PPO	75–80 %HRR	
	80–90 %VO_2_peak	100 %VT2 or %RCP	70–80 %VO_2_peak	

*MICT, moderate intensity continuous training; HIIT, high intensity interval training; HR, heart rate; HRR, heart rate reserve; RPE, rating of perceived exertion on 6-20 Borg scale (19); VO_2_, oxygen uptake; VT1, first ventilatory threshold; VT2, second ventilatory threshold; PPO, peak power output; RCP, respiratory compensation point*.

#### Influence of Exercise Intensity on CVD and Mortality

Numerous studies investigating all-cause mortality in healthy populations, have demonstrated that higher intensity exercise may induce larger health benefits than low or moderate intensity exercise. Furthermore, benefits with high intensity exercise can be achieved in substantially less time than MICT. The Hunt Study (60) demonstrated that one single bout of high intensity exercise reduced all-cause and CV mortality to a similar or greater degree than several hours of MICT. Similarly, Wen et al. (61), demonstrated the superior or time-efficient advantages of vigorous intensity exercise, with similar health benefit to MICT in half the weekly exercise time, or double the health benefit to MICT with the same weekly exercise time. Furthermore, studies have shown the proportion of vigorous activity has an inverse dose-response relationship with all-cause mortality in people with and without CVD, calling for physical activity (PA) guidelines to endorse participation in vigorous activity (62, 63). Finally, several studies have also shown an inverse association between exercise intensity and incidence of coronary heart disease in men independent of total exercise volume (64, 65), however the association of exercise intensity is less clear in women (66, 67).

#### Influence of Exercise Intensity on Cardiorespiratory Fitness

Cardiorespiratory fitness (assessed as VO_2_peak) reflects an integrated ability to transport oxygen (O_2_) around the body, encompassing pulmonary function, cardiac function (systolic and diastolic), ventricular-arterial coupling, vascular function, and the ability of muscle cells to receive and use O_2_ (68). There is extensive evidence that VO_2_peak is a strong predictor of future CV events and mortality, and even modest increments in VO_2_peak can be clinically meaningful in patients with CAD and HF (69–71). A landmark study by Kavanagh et al. in 12,169 CAD patients referred for CR, found that each 1.0 mL/kg/min increment in VO_2_peak was associated with a 9 % increase in survival. Moreover, Keteyian et al. (72) found an increased survival of 15 % per 1.0 mL/kg/min increment of VO_2_peak in patients with CAD. A study by Mikkelsen et al. (69) including 1,561 cardiac patients (predominately with CAD; 84 %), found that for every 1.0 mL/kg/min improvement in VO_2_peak during CR, there was a 21 % reduction in CV events and a 13 % reduction in all-cause mortality. In patients with HF, the HF-ACTION trial (71) showed that every 6 % improvement in VO_2_peak (adjusted for other factors) was associated with an 8 % lower risk of CV mortality and HF hospitalization, and a 7 % lower risk of all-cause mortality. In a large meta-regression analysis examining 55 trials of either HIIT or MICT compared with control in patients with CAD and HF, Uddin et al. (73) demonstrated that exercise intervention intensity was the greatest predictor of VO_2_peak post CR, even when including age, sex, and baseline fitness level in the multivariable regression model. Furthermore, each 10 % increase in exercise intensity (as %VO_2_peak or %HRpeak) was associated with a 1.0 mL/kg/min increase in VO_2_peak post CR. This is supported by Mitchell et al. (74) in patients attending CR for any indication, finding the greatest improvements in VO_2_peak with vigorous intensity exercise (5.5 mL/kg/min), followed by moderate-vigorous intensity (4.9 mL/kg/min), and then moderate intensity exercise (4.1 mL/kg/min). In contrast, a meta-analysis in patients with HF that adjusted for total exercise expenditure (75) found duration and frequency of exercise sessions to be greater predictors of VO_2_peak improvement than exercise intensity, however this review excluded interval training studies (including HIIT). Another meta-analysis in patients with HF that included HIIT studies (76), found high intensity (≥90 %HRpeak or ≥85 %HRR) but not vigorous intensity exercise (70–90 %HRpeak; 60–85 %HRR) produced larger improvements in VO_2_peak compared with moderate intensity exercise (55–80 %HRpeak; 50–60 %HRR) with gains of 3.3, 2.3, and 2.2 mL/kg/min, respectively. Low intensity exercise (40–55 %HRpeak; 20–40 %HRR) produced the smallest improvement (1.0 mL/kg/min) (76).

Studies based in the United Kingdom (UK) have found smaller improvements in cardiorespiratory fitness [~0.7–0.8 metabolic equivalents (METs)] compared with international programs (~1.5 METs) (77). The UK guidelines for CR programs typically recommend exercise of a moderate intensity (40–70 %HRR), compared with moderate-high intensity exercise recommendations in Canada (40–85 %HRR), United States (40–80 %VO_2_peak) and other European countries (40–80 %VO_2_peak; up to 90 %HRpeak) (14). Furthermore, a UK study by Nichols et al. (13) reported that the peak exercise training intensities achieved (46–54 %HRR) did not progress to the upper range of the UK exercise prescription targets (40–70 %HRR), and that after 8 weeks the exercise duration achieved (23 min) only marginally exceeded the minimum recommended duration of 20 min. Therefore, lower exercise intensity and volume have been reported as contributors to the smaller VO_2_peak improvements in CR programs within the UK (13, 78). This may also be typical of CR programs in other countries with low-moderate intensity guidelines, with potential to reduce the overall effectiveness of CR.

There have been several meta-analyses comparing HIIT with MICT on cardiorespiratory fitness as VO_2_peak. Weston et al. (51) examined 10 studies in patients with cardiometabolic disease and found that HIIT improved VO_2_peak by 19 % compared to 10 % with MICT (mean difference = 3.0 mL/kg/min). In patients with CAD, several meta-analyses have shown a superior effect of HIIT compared with MICT on VO_2_peak improvement, with a mean difference 1.3–1.8 mL/kg/min (52, 79–82). Pattyn et al. found HIIT protocols that were isocaloric with MICT were more likely to show superiority over MICT (+2.1 mL/kg/min) compared with HIIT protocols that were a lower energy expenditure to MICT (+0.2 mL/kg/min) (52). Furthermore, Way et al. (83) demonstrated that although women tend to experience lower absolute improvements in VO_2_peak with HIIT than men, they have similar relative improvements in VO_2_peak (83)_._ The FITR Heart Study, a recently published pragmatic trial in 93 CAD patients using RPE as the primary method of exercise prescription (36), also demonstrated a superior effect of HIIT compared with MICT during a 4-week CR program, with a mean difference in VO_2_peak improvement of 1.7 mL/kg/min. In contrast to this trial and previous meta-analyses, the SAINTEX-CAD multicenter trial in 200 patients with CAD, found both HIIT and MICT produced equally substantial improvements in VO_2_peak (23 and 20 %, respectively) during a 12-week CR program (84). A noteworthy consideration with this trial, was the higher training intensity of the MICT group (average training intensity of 80 %HRpeak), compared with previous trials that prescribed training intensity at 65–75 %HRpeak (42, 85). Although the SAINTEX-CAD had designed MICT to be prescribed at 70–75 %HRpeak, patients were not restrained from exercising at higher intensities. Therefore, results of the SAINTEX-CAD study suggest that continuous training may be equally effective to HIIT for improving VO_2_peak, when performed at a vigorous intensity. In patients with HF, meta-analyses have also found a superior effect of HIIT compared with MICT on VO_2_peak improvement, with a mean difference 1.0–2.4 mL/kg/min (52, 82, 86, 87). However, the large multicenter SMARTEX HF study (88) in 261 patients with HFrEF, found similar improvements in VO_2_peak with HIIT (1.4 mL/kg/min) and MICT (0.8 mL/kg/min) compared with an exercise guidelines group that showed a decrease in VO_2_peak (−1.0 mL/kg/min). While median training intensity for HIIT was 90 %HRpeak (interquartile range 88–92 %) and 77 %HRpeak (interquartile range 74–82 %) for MICT, 51 % of HIIT patients exercised at a lower intensity than prescribed and 80 % of MICT participants trained at a higher intensity than the protocol prescribed (88). In 180 patients with preserved ejection (HFpEF), the multicenter OptimEX-Clin study (89) found neither HIIT or MICT met the prespecified minimal clinically important improvement in VO_2_peak (2.5 mL/kg/min) compared with a PA guidelines control group. However, HIIT and MICT showed similar increases in VO_2_peak of 1.1 and 1.6 mL/kg/min, respectively, over the 3-month supervised training (89). The meta-analysis by Pattyn et al. (52), also found no differences between HIIT and MICT for improvement of VO_2_peak in the HFpEF sub-analysis.

#### Influence of Exercise Intensity on Other Cardiometabolic Parameters

Exercise intensity appears to have a significant influence on increasing exercise capacity at VT1. An early study by Jensen et al. in patients with CAD (90), found high intensity continuous training was superior to low intensity training for improving VO_2_ at VT1. Furthermore, the meta-analysis by Pattyn et al. (52) in both CAD and HF patients, found greater improvements in VO_2_ at VTI after HIIT compared with MICT (mean difference of 0.9 mL/kg/min). This is important as the improved ability to use O_2_ aerobically may translate into improved performance of daily living activities (52). Pattyn et al. (52) also found a greater improvement in HRpeak with HIIT compared with MICT, and a trend for greater improvement in peak O_2_ pulse and O_2_ uptake efficiency slope (OUES) favoring HIIT. Other cardiorespiratory parameters (e.g., HR recovery, VE/VCO_2_ slope) and CVD risk factors (e.g., body weight, resting HR, blood pressure, cholesterol, triglycerides, and fasting glucose) do not appear to be influenced by exercise intensity (52, 82, 91). Studies investigating 24-hr blood pressure, have found a superior effect of HIIT compared with MICT in patients with hypertension (92) but similar improvement to MICT in patients with HF (93). Pattyn et al. (52) found a trend (*p* = 0.09) toward greater improvements in vascular function [via flow-mediated dilation (FMD)] with HIIT compared with MICT. In a meta-analysis with a more diverse cohort of cardiometabolic diseases, Ramos et al. (94) found HIIT was superior to MICT with a 2-fold greater improvement in flow-mediated dilation (4.31 vs. 2.15 %, respectively). For changes in body composition, HIIT provides similar benefit compared with MICT, but not when total energy expenditure is less (95). Therefore, exercise volume appears to play a greater role in body composition than exercise intensity (95). A retrospective study by Dun et al. (96) in 120 CR patients with myocardial infarction, found greater reductions in total fat mass and abdominal fat percentage with HIIT compared to MICT using dual-energy x-ray absorptiometry (DEXA). In contrast, Taylor et al. (97) found similar reductions for visceral adipose tissue and subcutaneous fat quantified by magnetic resonance imaging and total fat mass with DEXA, when comparing isocaloric HIIT and MICT in patients with CAD. The influence of exercise intensity on resting left ventricular ejection fraction (LVEF) and left ventricular end-diastolic diameter (LVEDD) remain inconclusive. Meta-analyses by Cornelis et al. (98) and Pattyn et al. (52) both found HIIT significantly improved both LVEF compared with MICT in patients with HF, however Haykowsky et al. (86) found only a trend toward greater improvements in LVEF. Cornelis et al. (98) also found HIIT significantly improved LVEDD compared with MICT. While the multi-center SMARTEX HF study (88) found only HIIT significantly improved LVEDD after 12-weeks compared with the control group, there was no difference between HIIT and MICT.

#### Influence of Exercise Intensity on Long-Term Outcomes and Adherence

Only three studies have investigated long-term outcomes of HIIT compared with MICT in patients with CAD at 6-months (85) and 12-months (36, 99). Moholdt et al. (85) found a superior effect of HIIT compared with MICT on improvement of VO_2_peak and HR recovery at 6-months in patients with CABG, but similar improvements in quality of life and adiponectin. At 12-months, the SAINTEX-CAD and FITR Heart studies found similar improvements between HIIT and MICT in patients with CAD for VO_2_peak and other exercise variables (36, 99), CVD risk factors (36, 99), quality of life (36, 99), FMD (99, 100), body composition (97, 101), moderate-vigorous PA (36, 99), and no changes in dietary intake (101). Although for The FITR Heart Study, the improvement in VO_2_peak was numerically higher for HIIT (2.9 mL/kg/min) than MICT (1.8 mL/kg/min) (36) which may be related to greater long-term survival as noted above. In contrast, the SMARTEX HF (88) and OptimEX-Clin (89) studies in patients with HFrEF and HFpEF, respectively, showed a regression of supervised training improvements at 12-months regardless of exercise intensity. A difference with The FITR Heart Study (36) and OptimEx-Clin study (89) was after the supervised training period, participants were instructed to continue home-based HIIT or MICT (as randomized) until the 12-month follow-up, and therefore long-term adherence to the HIIT and MICT protocols were assessed. At 12-months, The FITR Heart Study reported 38 % of MICT participants had starting exercising at a higher intensity than prescribed and 24 % of HIIT participants exercised at a lower intensity than prescribed, although overall adherence (>70 % of sessions at prescribed intensity) was similar between groups (53 % for HIIT and 41 % for MICT) (36). When non-adherent participants were excluded from the analysis, HIIT showed a considerably greater improvement in VO_2_peak (5.2 mL/kg/min) compared with MICT (2.2 mL/kg/min) (36), however improvements in other cardiometabolic outcomes remained similar between groups. This demonstrates that adherence to the intensity of exercise protocols over the long-term can significantly influence improvements in VO_2_peak. In contrast, Moholdt et al. (85) found after 5 months of home-based training that a higher proportion of participants stopped HIIT in favor of MICT (35 %), compared to only 4 % of MICT participants starting higher intensity exercise. Although adherence to the randomized training (≥3 times/week) at 6-months was slightly lower for HIIT (52 %) than MICT (64 %), the proportion of participants performing 3 sessions/week of at least moderate intensity exercise was similar for HIIT (74 %) and MICT (68 %), and improvements in VO_2_peak were superior with HIIT at 6-months. In patients with HFpEF, the Optimex-Clin study (89) reported that adherence (>70 % of sessions) did not influence improvements in VO_2_peak. The authors found similar adherence between HIIT and MICT (56 and 60 %, respectively) but did not report on adherence to the intensity of the exercise protocols. A recent review in CR patients with CAD, found short-term adherence (as number of sessions) to supervised or home-based HIIT was similar to MICT (53). However, the review highlighted that adherence to intensity and duration of the training protocols was under-reported, and the authors provided recommendations for how future studies can collect and report this important data (53). This is particularly important given the findings from larger pragmatic trials such as the SAINTEX-CAD, SMARTEX-HF, and FITR Heart studies on non-adherence to training intensity, which can provide insight into feasibility and effectiveness of exercise prescription. The review by Taylor et al. (53) in patients with CAD, found the majority of studies reporting on feasibility (8/11 studies) reported HIIT to be equally feasible to MICT in patients attending CR, while the other three studies reported HIIT was less feasible than MICT. Factors that appeared to improve feasibility of HIIT included: setting realistic expectations for training intensities; including a variety of exercise modalities (for enjoyment and reducing musculoskeletal impact); and using progressive models of HIIT (53).

### Physiological Adaptations With Exercise Training for Improving VO_2_peak

**Figure 1:**
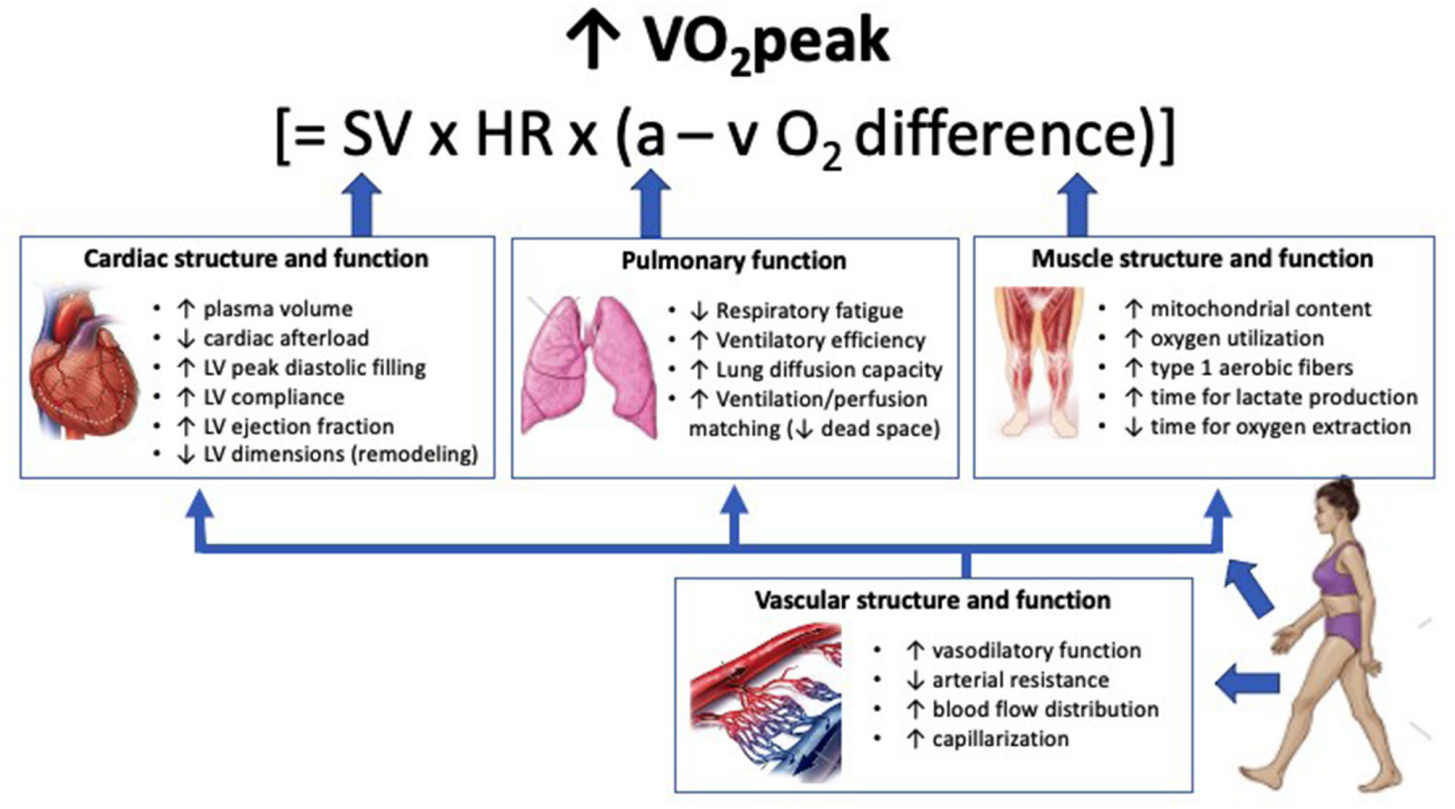
Physiological adaptations from exercise training that may contribute to improvement in VO_2_peak in patients with cardiovascular disease or heart failure. Cardiac adaptations contribute to VO_2_peak improvements primarily through increases in stroke volume. Pulmonary adaptations contribute to VO_2_peak by optimizing arterial oxygen content and therefore oxygen delivery. Muscle adaptations contribute to VO_2_peak improvements through increased exercise tolerance and enhanced oxygen extraction. Vascular adaptations contribute to oxygen delivery by reducing arterial resistance, increasing capillary density, and enhancing blood flow distribution, which in turn enhances function of the cardiac, pulmonary, and muscle systems. Increases in plasma volume can occur within days of commencing exercise training. Adaptations within skeletal muscle and to the vasculature can occur within weeks of training. Current evidence supports greater adaptations in mitochondrial content, vascular vasodilatory function, and stroke volume following HIIT compared with MICT, although the precise cardiac mechanisms that lead to increased stroke volume remain unclear. VO_2_peak, peak oxygen uptake; SV, stroke volume; HR, heart rate; a–vO_2_ difference, oxygen content difference between arterial and venous blood; LV, left ventricle.

There are numerous integrative physiological adaptations that may improve VO_2_peak in patients with CVD or HF (Figure 1). Based on the Fick equation, VO_2_ is the product of cardiac output and arterial-venous O_2_ content difference (a-vO_2_ difference), where cardiac output is the product of stroke volume and HR (68). This equation can also be summarized as “central” and “peripheral” determinants of VO_2_, respectively (50).

#### Pulmonary Adaptations

The pulmonary system is responsible for the transport of O_2_ from the atmosphere to the bloodstream, with alveolar ventilation (O_2_ exchange with the atmosphere) and diffusion (O_2_ exchange with the bloodstream) contributing to arterial O_2_ content, O_2_ delivery, and VO_2_peak (68). Reduced alveolar exchange can be a significant contributor to exercise intolerance in patients with HF, which can occur due to impaired pulmonary vasodilation, ventilation/perfusion mismatch, impaired diffusion, abnormal ventilatory reserve (i.e., respiratory muscle dysfunction), or abnormal ventilatory regulation (i.e., oscillatory patterns) (102–105). Furthermore, respiratory muscle fatigue has also been shown to affect O_2_ delivery by causing peripheral vasoconstriction and reduced blood flow to skeletal muscles, further exacerbating exercise intolerance (106). There are limited studies comparing the effect of exercise training intensity on pulmonary adaptations. In patients with HF, Tasoulis et al. (107) demonstrated that HIIT improved ventilatory regulation, with an improvement in ventilatory drive (as P_0.1_/PI_max_) and ventilatory patterns during rest and exercise, although there was no control group. In healthy adults, Dunham and Harms (108), demonstrated significant improvements in respiratory muscle function with HIIT and MICT (43 vs. 25 %) over 4-weeks, however there was a greater increase with HIIT. Finally, Guazzi et al. (109) studied patients with HF, and found that compared with a control group, 8-weeks of moderate-vigorous intensity training improved lung diffusion, alveolar-capillary conductance, and pulmonary capillary blood volume with concomitant improvements in VO_2_peak. Whether HIIT is superior to MICT for pulmonary adaptations remains unclear.

#### Cardiac Adaptations

One of the proposed mechanisms for greater improvement in VO_2_peak with higher intensity exercise is greater central adaptations in left ventricular structure and function (110), by challenging the cardiac muscle to provide increased cardiac output and O_2_ to the working muscles (52). In athletes, exercise training is associated with expansion of red blood cell volume and augmented plasma volume (111), reduced total peripheral resistance, and increased LV end-diastolic volumes (i.e., LVEDV), leading to increased stroke volume, cardiac output, and VO_2_peak (112). Increased LV diastolic volumes in the short-term can be attributed to increased plasma volume and venous return via the Frank-Starling mechanism (111), while long-term adaptation involves structural changes from exercise training that enhance LV compliance (112). However, in cardiac patients, who may have pathological LV dilation (cardiomegaly), exercise training has been associated with reversal of LV remodeling (i.e., reduction in LVEDD), increases in peak diastolic filling, and reduced peripheral resistance, with concomitant increases in stroke volume, cardiac output, and VO_2_peak (44, 113, 114). Several studies in healthy subjects (115, 116) and patients with HFrEF (44, 58, 117) have shown greater improvements in maximal stroke volume alongside VO_2_peak with HIIT compared with MICT. In contrast, Iellamo et al. (118) found no improvement in central hemodynamics (cardiac output or stroke volume) for HIIT or MICT in patients with HFrEF despite large increases in VO_2_peak for both groups. A proposed reason for differences in central adaptation outcomes between studies, is that some patients with a high degree of peripheral limitation (e.g., muscle atrophy or overt cachexia) may have a limited ability to exercise at a high intensity for a sufficient amount of time (110). Peripheral limitations are known to be a significant contributor to exercise intolerance in patients with HF (102). Inability to achieve the target training intensity of HIIT (51 % participants) was a significant limitation of the SMART-EX HF study in patients with HFrEF (88), which showed no change in LVEDD or peak O_2_ pulse (a surrogate for stroke volume). In contrast, the SAINTEX-CAD study in patients without HF (84) showed significant improvements in peak O_2_ pulse for both HIIT and MICT, although the average intensity of the MICT group was 80 %HRpeak and therefore higher than a moderate intensity. In summary, majority of studies investigating central hemodynamics have found greater improvements in stroke volume with HIIT compared with MICT. However, the effect of exercise training intensity on structural adaptations remains unclear.

#### Vascular Adaptations

Another potential mechanism for the greater improvement in VO_2_peak with higher intensity exercise is the superior effect on vascular function for HIIT compared with MICT (52, 94). Greater elasticity and function of the central and peripheral vasculature allows for greater accommodation and more efficient transport of blood and O_2_ to the heart and skeletal muscles (68). A common method for measuring vascular function adaptations is brachial artery FMD (119), a non-invasive test shown to correlate well with invasively measured coronary artery vasodilatory function (120). There is extensive evidence that aerobic exercise training improves vascular function in large conduit arteries, with repeated hemodynamic stimuli and laminar shear stress playing a central role in vascular adaptation (119). A proposed mechanism for the superior effect of HIIT on vascular function, when compared with MICT, is that higher intensity exercise provokes greater blood flow and shear stress stimulus, that allows for greater vascular adaptation through upregulation of vasodilatory prostaglandins (119, 121) and nitric oxide (119, 122). Thijssen et al. (123) demonstrated that with incremental increases in exercise intensity for various modalities (walking, cycling, leg kicking), there was a parallel increase in mean blood flow and shear rate within the brachial artery.

#### Skeletal Muscle Adaptations

In addition to O_2_ delivery, peripheral adaptations with training that increase O_2_ extraction and utilization may also lead to increases in VO_2_peak by increasing a-vO_2_ difference. In healthy populations, skeletal muscle capacity for oxidation usually exceeds the capacity for systemic O_2_ transport (50), and therefore peripheral adaptations may not contribute to large increases in VO_2_peak. However, in deconditioned patients, particularly those with HFrEF and HFpEF, peripheral adaptations within skeletal muscle can have a significant effect on exercise tolerance and capacity (102, 124). The major training-induced adaptations that increase O_2_ extraction and utilization within skeletal muscle, include increased capillary density and mitochondrial volume density (111). The former may enhance local blood perfusion and distribution with or without improvements in vascular function (111), while the latter increases capacity for substrate oxidation at a given workload (50, 125). Increased mitochondrial content with exercise training “promotes greater reliance on fat oxidation with a proportional decrease in carbohydrate oxidation,” which in turn “reduces glycogen degradation and lactate production at a given workload” (126, 127). As a result, lactate threshold is increased and patients can exercise for longer durations at a greater percentage of VO_2_peak (128). This is particularly important for cardiac patients, as reduced oxidative capacity can significantly contribute to exercise intolerance (102). There is strong evidence from studies in healthy populations that exercise intensity mediates mitochondrial adaptations to exercise and improvements in VO_2_peak (126). During higher intensity exercise, there is greater accumulation of metabolites and free radicals from calcium release, ATP turnover, and carbohydrate utilization (1, 126). This accumulation leads to activation of several mitochondrial enzymes, which stimulate expression of peroxisome proliferator-activated receptor γ coactivator-1*a* (PGC-1*a*), an important regulator of mitochondrial biogenesis (50, 126). Studies involving patients with HF (44), metabolic syndrome (43), and obesity (129) have shown greater increases in PGC-1*a* with HIIT compared with MICT, with concomitant increases in the maximal rate of calcium reuptake into the sarcoplasmic reticulum. Enhanced calcium cycling, may also help to reduce muscle fatigue and exercise capacity (51). In addition to exercise intensity, this greater activation of signaling pathways for mitochondrial adaptations is thought to be triggered by the metabolic fluctuations with intermittent exercise bouts, that occurs during interval training (50). In contrast, short bursts of high intensity exercise do not appear to improve vascular endothelial growth factor (VEGF) secretion and skeletal muscle capillarization, which may require longer durations of constant work exercise (130). Studies that have found superior improvements in VEGF and skeletal muscle capillarization with MICT compared with HIIT, have used short duration HIIT intervals of ≤1min (131, 132). There is limited research comparing training of different intensities on muscle fiber type. However, studies comparing exercise to a control group, have found an increased proportion of type 1 fibers with decreased type IIb fibers after either 15-weeks of SIT (133) or 6-months of MICT (134). On the other hand, Tan et al. (135), found 6-weeks of HIIT improved oxidative capacity of both type 1 and type 2 muscle fibers to a similar degree. Based on the current evidence, it appears that high intensity exercise provides a potent stimulus for mitochondrial adaptations compared with MICT, however the effect of training intensity on other peripheral adaptations such as capillarization, blood flow distribution, and muscle fiber shift, remain unclear.

#### Influence of Interval Duration and Protocol Volume on Physiological Adaptations

Currently, there is no consensus on the optimal HIIT protocol. Moreover, the effectiveness of HIIT protocols may vary according to the physiological adaptation of interest. HIIT protocols have previously been classified by interval duration as short-duration (≤1 min), medium-duration (1–3 min), or long-duration (≥3 min) (136, 137). Furthermore, high-volume HIIT has been defined as protocols that accumulate ≥15 min of high intensity effort per session (138). The Norwegian model (110) involving 4 × 4min high intensity intervals (85–95 %HRpeak) separated by 3 min active recovery intervals, is an example of a long-duration, high-volume HIIT protocol, that has been studied extensively in populations with CAD and HF. Two meta-analyses in healthy populations suggest that longer interval durations increase VO_2_peak to a greater extent than short duration intervals (139, 140). This may be related to greater improvements in central adaptations with longer duration intervals. This is supported by Matsuo et al. (116), who compared SIT (7 × 30 s intervals; 100 kcal), HIIT (3 × 3 min intervals; 180 kcal) and MICT (40 min; 360 kcal), and found the greatest increases in stroke volume, LV mass, and VO_2_peak in the HIIT group, followed by the SIT group. Therefore, exercise intensity is important, but also time to reach and maintain an elevated cardiac filling (which can take 1–4 min in athletes) is believed to be necessary for improving maximal cardiac function (41). However, in the meta-analysis by Pattyn et al. in patients with CAD and HF (52), subgroup analyses revealed no differences in VO_2_peak improvement based on the duration of the HIIT intervals. Instead, intensity of the HIIT intervals appeared more important, with numerically larger increases in VO_2_peak with HIIT intervals at a very hard near-maximal effort (+1.5 mL/kg/min) compared with HIIT intervals at a vigorous effort (+1.1 mL/kg/min) (52). Moreover, HIIT protocols with greater total energy expenditure also produced greater gains in VO_2_peak (52). This is supported by others, in that high-volume HIIT protocols appear to elicit the greatest increases in VO_2_peak (138, 140) and vascular function (94).

### Practical Application and Progression Models for HIIT in CR Programs

#### Safety Considerations

While HIIT provides greater improvements in VO_2_peak, there remains a concern regarding its safety in cardiac populations (91). A scientific report from the American Heart Association (AHA) (141), outlined that vigorous exercise can acutely and transiently increase risk of sudden cardiac death and acute myocardial infarction in patients with atherosclerotic disease. However, this report and others have highlighted that incidence of these events is greatest in adults who are the least active (141, 142). For deconditioned patients, many of their daily living activities can fall into the category of vigorous intensity (143). Concerns around safety should take this into account, as including HIIT with appropriate progression may expose patients to vigorous efforts in a safer and more controlled manner. The most recent systematic review and meta-analysis on safety of HIIT in patients with CVD, found that HIIT showed a low rate of major adverse events for patients with CAD and HF when applied in CR settings (144). As all studies within the review had included baseline exercise testing, this was a recommendation from the authors prior to HIIT (144). However, maximal exercise testing is not routinely conducted in many CR settings, although guidelines in North America and Europe do recommend electrocardiographic exercise testing as standard procedure (14). The FITR Heart Study medically excluded 3 % of participants following baseline exercise testing, however the need for further coronary intervention was very low (1 %) (36). The AHA report (141), also outlines that appropriate screening and exclusion of high-risk patients from vigorous activities, can help to minimize the incidence of CV events. To assist clinicians in safely implementing HIIT without maximal exercise testing, guidelines have been published on screening and monitoring for HIIT in clinical populations (26). For example, three studies have reported a hypotensive event during HIIT (36, 88, 145). Therefore, in patients taking anti-hypertensive medication, a gradual and extended cool-down is recommended, particularly if medications have been recently modified (26). Given the higher risk of CV events with vigorous activity in adults who are less physically fit and active (141, 142), starting patients with a lead-in period of MICT is a sensible approach to ensure proper education on exercise training, assess exercise response, improve exercise tolerance, and minimize musculoskeletal injuries, particularly for patients who are unaccustomed to vigorous exercise (144). Furthermore, progressively increasing interval duration and time spent at a vigorous intensity (see practical applications section), may improve safety and exercise tolerance. Recent guidelines from the European Society of Cardiology (146) outline that high intensity exercise is appropriate for low risk revascularized patients with CAD, if they are asymptomatic and stable, and without residual high risk CAD lesions or exercise-induced arrhythmias. For patients with HF (reduced and preserved ejection fraction), high intensity exercise can also be prescribed for patients who are stable and without exercise-induced arrhythmias (146). Further studies are required to determine whether high intensity exercise is safe in higher risk patients with CAD or HF.

#### Intensity Prescription of HIIT

As outlined earlier in this review, all methods of exercise prescription have their advantages and limitations in patients with CVD and HF. For this reason, we recommend using both objective and subjective measures to prescribe exercise intensity for HIIT. The multicenter SAINTEX-CAD study relied only on objective measures of intensity for HIIT prescription, and subsequently found and acknowledged that when prescribing HIIT “it is necessary to adjust the objectively defined target HR zones and workloads according to the patient's subjective feelings.” Furthermore, the SAINTEX-CAD study (84) and Pattyn et al. (52) have found significant increases in HRpeak over 12-weeks of training. This suggests that target HR zones may need to be adapted over the training period, either by repeating a maximal exercise test, or using subjective measures of intensity to titrate the workload accordingly. Patients may experience the same external training load (e.g., %HRpeak) differently depending on their individual “internal” metabolic responses to changes in exercise intensity (e.g., lactate accumulation) (147). Therefore, subjective measures are important to consider, and RPE has been shown to be a good indicator of internal training load (37). Currently the most common methods for prescribing HIIT that are also practical for clinical settings, are %HRpeak and RPE. For shorter duration intervals (<2 min), %HRpeak may underestimate the training stimulus due to insufficient time for HR to rise and HR lag compared with VO_2_ response (41, 53), particularly in patients with HF or chronotropic incompetence. For long-duration HIIT, a framework for clinicians on using a combination of %HRpeak and RPE for HIIT prescription has previously been outlined by Taylor et al. (26). This framework involves using a maximally-derived or estimated HRpeak to determine a training target of 85–95 %HRpeak, in combination with a validation session with RPE of 15–18 by the patient, or observer (clinician supervising the exercise) if a patient has difficulty reporting an accurate RPE. Although it is practically attractive for clinicians to solely use RPE for HIIT prescription, Aamot et al. (148) found that using an RPE of 17 (very hard) alone for HIIT prescription, results in a lower mean training intensity (82 %HRpeak) than a target range of 85–95 %HRpeak. Therefore, HR monitoring in combination with RPE during HIIT may result in greater adherence to exercise intensity targets that are optimal for HIIT (85–95 %HRpeak).

#### Incorporating HIIT Into the Optimal Exercise Dose

Exercise volume, or “dose” encompasses both exercise intensity and duration of exercise (21). According to current PA guidelines from the World Health Organization (149) and US Department of Health (150), it is recommended that adults (even those with chronic health conditions) should accumulate 150–300 min/week of moderate intensity exercise, or 75–150 min/week of vigorous intensity exercise, or an equivalent combination of moderate and vigorous exercise. In the context of high-volume HIIT protocols that typically involve ~16 min/session of high intensity effort and 10–15 min/session of moderate intensity effort, three sessions/week of high-volume HIIT would provide ~48 min/week of vigorous exercise and ~45 min/week of moderate exercise. While this may approach the minimum level of the PA recommendations, guidelines advocate for achieving more than the minimum level of PA to sustain optimal health (149, 150). Moreover, it is recommended that patients undergoing CR, progress to an optimal weekly exercise dose equivalent to 1,500 kcal/week (151, 152). For example, a high-volume HIIT protocol in patients with CAD measured energy expenditure to be ~50L O_2_/session (42) [equating to ~250 kcal/session with ACSM estimation of 1L VO_2_ = 5 kcal (21)]. Therefore, three sessions/week would equate to half the weekly exercise dose that patients undergoing CR should progress to (1,500 kcal/week) (151, 152). To extend the volume of training beyond the minimum level of PA recommendations and progress to 1,500 kcal/week, while also allowing for variety of exercise training, HIIT can be prescribed as an adjunct rather than alternative to MICT. For example, HIIT could be prescribed as 3 sessions/week in combination with 2–3 sessions/week of MICT and/or resistance training. Alternating days of HIIT and MICT training may also aid in recovery from HIIT sessions while reducing the potential for musculoskeletal discomfort.

Exercise volume can also be quantified as MET-min per week, which is calculated as intensity (in METs) multiplied by the number of minutes at that intensity, accumulated over a week. Current PA guidelines recommend 500–1,000 MET-min per week from moderate-vigorous activities (153). According to ACSM (49), average METs for moderate intensity exercise ranges from 3.0 to 5.9 METs, vigorous intensity exercise ranges from 6.0 to 8.7 METs, and near-maximal intensity exercise is ≥ 8.8 METs, although these MET ranges can vary according to age. For use in clinical practice, METs can be estimated from treadmill and cycle workload equations (21, 154, 155), a list of PA intensities (156), and/or some commercial exercise equipment can provide an estimate of METs.

In addition to energy expenditure, methods have been explored that consider individual “internal” training responses to quantify and monitor training dose (147). For example, the training impulse (TRIMP) is calculated by multiplication of (1) the duration of a specific training session, (2) the average change in HR (i.e., HRexercise–HRrest/HRmax–HRrest) during the training session, and (3) an individual weighting factor to reflect metabolic effort (157). This weighting factor is calculated from a maximal exercise test as the best-fitting exponential line from a plot of blood lactate concentration against fractional elevation in HR (157). Given the potential complexities of this method in a clinical setting (i.e., availability of maximal exercise testing, blood lactate measurement, and limitations with HRmax), a session-RPE method (i.e., RPE representative of the overall session multiplied by session duration), has been validated in patients with HF as an alternative to TRIMP for monitoring training dose (37). However, further research is needed to determine the optimal weekly session-RPE for CR programs.

#### Interval Duration and Progression of HIIT

Although high-volume HIIT with longer-duration intervals may increase exercise dose and provide superior improvements in central adaptations, vascular adaptations, and VO_2_peak, sustaining high intensity of exercise for longer than 1–2 min may be challenging for some patients commencing a CR program. In particular, patients who are exercise naïve (137) and/or have a high degree of exercise intolerance (from skeletal muscle dysfunction, respiratory limitations, reduced cardiac reserve, or a combination of these factors) (102), would benefit from a more gradual introduction to HIIT. Primary components of exercise prescription defined by ACSM include frequency, intensity, time, type, volume, and progression (FITT-VP) (21). Progression can often be a difficult component of exercise prescription for clinicians to master, but essential to optimize gains in VO_2_peak and minimize adverse complications (151). In athletic and healthy populations progression has traditionally involved the training principles of progressive overload, specificity, and periodization (136). It seems appropriate that throughout a CR program, patients may undergo progression from short-duration intervals, to medium-duration intervals, and finally to long-duration intervals as training-induced physiological adaptations occur and exercise tolerance improves (137, 158). Overload is defined as “an exercise dose which is above and beyond the accustomed amount of exercise for a given individual” (151). For aerobic training, it is generally recommended to just increase one component of frequency, intensity, or duration at a time (151). As suggested by Wewege et al. (144), commencing a CR program with a “lead-in period” of MICT seems appropriate before commencing HIIT. This may allow for a graduated approach to evaluate a patient's exercise response, improve exercise tolerance, and aid in minimizing adverse events and musculoskeletal injuries (144). Furthermore, in terms of progression, it is generally recommended in cardiac patients that “duration and frequency of exercise should be up-titrated before intensity is increased” (to ≥30 min/session, 4 days/week) (159, 160). Once patients are tolerating 30 min of MICT, intensity could then be progressed to include short-duration HIIT, which may provide a greater stimulus than MICT for improving mitochondrial volume and oxidative capacity (50). Adaptations to mitochondrial content have been shown to occur at a rapid rate, with as little as 6 sessions of HIIT in healthy populations (126). Further progressions in interval duration from medium-duration to long-duration could then be made throughout the CR program, to further improvements in central adaptations (116), vascular function (94), and VO_2_peak (138, 140). Another option for progression is to introduce HIIT once/week initially and then progress to 2–3 sessions/week.

#### HIIT Progression Model Example

**Figure 2:**
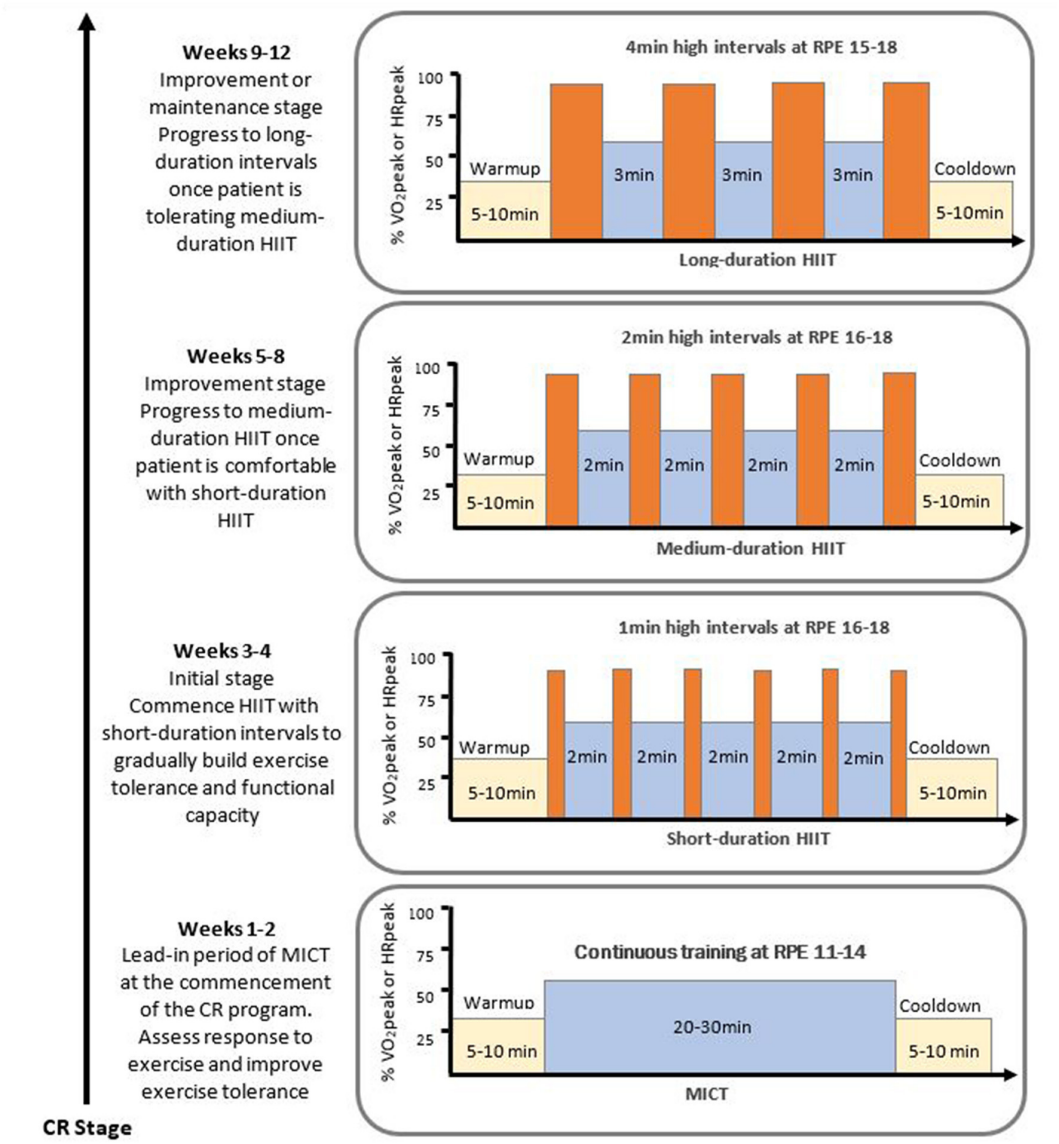
Example of a HIIT progression model within a cardiac rehabilitation program. Exercise intensity remains constant for each HIIT protocol with high intensity intervals eliciting 85-95 %HRpeak and RPE 15-18, and the low intensity intervals involving recovery at 50-75 %HRpeak or RPE 11-14. CR, cardiac rehabilitation; HIIT, high intensity interval training; HRpeak, peak heart rate; MICT, moderate intensity continuous training; RPE, rating of perceived exertion on 6-20 Borg scale; VO_2_peak, peak oxygen consumption. This figure has been adapted from the previously published work of (158); with permission of Mayo Foundation for Medical Education and Research, all rights reserved.

In Figure 2 we provide an example of how HIIT commencement and progression could occur during a CR program. Following a 2-week lead-in period of MICT, patients with low functional capacity [<5 metabolic equivalent (METs)] (161) or in the initial stage of HIIT, can commence a short-duration HIIT protocol (e.g., 1 min HIIT interval every 3–4 min of MICT), with progression to reduce the recovery interval timing to 2 min. As patients understanding of exercise training and comfort level with the available training modalities increases to an acceptable level, further progressions in interval duration to medium-duration intervals could be prescribed (e.g., 2–3 min HIIT with 2 min recovery). Initially, clinicians may want to keep the total time at high intensity constant (e.g., from 6 × 1 min HIIT to 3 × 2 min HIIT), and then gradually progress the number of intervals to 5 × 2 min or 4 × 3 min over a number of weeks by just changing one prescription factor (interval frequency or duration) at a time. Once patients are comfortable with 4 × 3 min intervals, patients could be progressed to 4-min intervals with 3 min recovery for a high-volume HIIT protocol (e.g., 4 × 4 min) to further improvements in central adaptations (116), vascular function (94), and VO_2_peak (138, 140). Once again, clinicians may want to initially keep the total time at high intensity constant (e.g., from 4 × 3 min HIIT to 3 × 4 min HIIT) and then progress the number of intervals to 4 × 4 min HIIT.

## Conclusion

There is extensive evidence that higher intensity exercise contributes to greater improvements in VO_2_peak than MICT or low intensity exercise, by increments that are known to be clinically meaningful. Higher intensity exercise also produces greater improvements in VO_2_ at submaximal exercise, which is important for exercise tolerance and carrying out daily living activities. While short-duration HIIT protocols can be a potent stimulus for improving peripheral mitochondrial adaptations and providing similar VO_2_peak improvements to MICT, longer-duration and higher-volume HIIT protocols seem to be superior for eliciting stroke volume and vascular adaptations, and greater VO_2_peak improvements compared with MICT. Finally, rather than adopting a one-size fits all model for HIIT, gradual introduction and progression of HIIT in accordance with individual exercise experience and tolerance, may be optimal for reducing musculoskeletal discomfort, as well as maximizing safety, adherence, enjoyment, and physiological outcomes.

## Author Contributions

JLT, ARB, and TPO all contributed to the conception of the manuscript idea and design. JLT was responsible for composing the manuscript. ARB and TPO provided critical revision of the manuscript. All authors contributed to the article and approved the submitted version.

## Conflict of Interest

The authors declare that the research was conducted in the absence of any commercial or financial relationships that could be construed as a potential conflict of interest.

## Publisher's Note

All claims expressed in this article are solely those of the authors and do not necessarily represent those of their affiliated organizations, or those of the publisher, the editors and the reviewers. Any product that may be evaluated in this article, or claim that may be made by its manufacturer, is not guaranteed or endorsed by the publisher.
